# The Intersection of NGF/TrkA Signaling and Amyloid Precursor Protein Processing in Alzheimer’s Disease Neuropathology

**DOI:** 10.3390/ijms18061319

**Published:** 2017-06-20

**Authors:** Nadia Canu, Giuseppina Amadoro, Viviana Triaca, Valentina Latina, Valentina Sposato, Veronica Corsetti, Cinzia Severini, Maria Teresa Ciotti, Pietro Calissano

**Affiliations:** 1 Department of System Medicine, University of Rome “Tor Vergata”, Via Montpellier 1, 00137 Rome, Italy; nadia.canu@uniroma2.it or nadia.canu@ibcn.cnr.it; 2 Institute of Cellular Biology and Neurobiology, National Council of Research Rome, Via del Fosso del Fiorano 64, 00143 Rome, Italy; viviana.triaca@ibcn.cnr.it (V.T.); cinzia.severini@cnr.it (C.S.); teresa.ciotti@ibcn.cnr.it (M.T.C.); 3 Institute of Translational Pharmacology, National Research Council (CNR) Rome, Via Fosso del Cavaliere 100, 00133 Rome, Italy; g.amadoro@inmm.cnr.it (G.A.); valentina.latina@libero.it (V.L.); 4 European Brain Research Institute Rome, Via del Fosso del Fiorano 64, 00143 Rome, Italy; v.sposato@ebri.it (V.S.); vcorsetti@libero.it (V.C.)

**Keywords:** nerve growth factor (NGF), Tropomyosin receptor kinase A (TrkA) receptor, basal forebrain cholinergic neurons (BFCN), Amyloid Precursor Protein (APP), synapses, tau protein, Alzheimer’s disease (AD)

## Abstract

Dysfunction of nerve growth factor (NGF) and its high-affinity Tropomyosin receptor kinase A (TrkA) receptor has been suggested to contribute to the selective degeneration of basal forebrain cholinergic neurons (BFCN) associated with the progressive cognitive decline in Alzheimer's disease (AD). The aim of this review is to describe our progress in elucidating the molecular mechanisms underlying the dynamic interplay between NGF/TrkA signaling and amyloid precursor protein (APP) metabolism within the context of AD neuropathology. This is mainly based on the finding that TrkA receptor binding to APP depends on a minimal stretch of ~20 amino acids located in the juxtamembrane/extracellular domain of APP that carries the α- and β-secretase cleavage sites. Here, we provide evidence that: (i) NGF could be one of the “routing” proteins responsible for modulating the metabolism of APP from amyloidogenic towards non-amyloidogenic processing via binding to the TrkA receptor; (ii) the loss of NGF/TrkA signaling could be linked to sporadic AD contributing to the classical hallmarks of the neuropathology, such as synaptic loss, β-amyloid peptide (Aβ) deposition and tau abnormalities. These findings will hopefully help to design therapeutic strategies for AD treatment aimed at preserving cholinergic function and anti-amyloidogenic activity of the physiological NGF/TrkA pathway in the septo-hippocampal system.

## 1. Introduction

Alzheimer′s disease (AD), a progressive and multifactorial neurodegenerative disease with both genetic and environmental causes, represents the most common form of dementia among the elderly (50–70% of late-life cases). The sporadic AD (SAD) (late onset >60 years) and the rare familial forms (FAD) (early onset <60 years) share many clinical and pathological features. Senile plaques (SP) and neurofibrillary tangles (NFT) result from the accumulation and deposition of β-amyloid peptide (Aβ) and the aggregation of hyperphosphorylated tau protein, causing deficits in the integrity and functions of synapses. Recent studies indicate that the pathological process begins decades before the onset of clinical signs, starting from subcortical nuclei innervating the hippocampus and the cortex, and subsequently spreading throughout the brain [1]. The basal forebrain cholinergic nuclei (BFCN) represents a brain area with a high susceptibility to AD, and is the major source of cholinergic innervation to the cerebral cortex and hippocampal regions involved in memory and attentional processes [2]. Of note, cholinergic dysfunction is already detectable at the preclinical stage of AD neuropathology, and progresses towards overt neurodegeneration in late stages of AD, paralleling typical AD cognitive deficits.

The selective death of BFCN can be counteracted by neurotrophins, of which Nerve Growth Factor (NGF) is the most potent in vitro and in vivo [3,4,5]. NGF transduces its effects by binding two classes of cell surface receptors, both of which are expressed within the BFCN. These are the high affinity tropomyosin-related kinase A (TrkA) receptor and the low-affinity p75 neurotrophin receptor (p75NTR). NGF produced by hippocampal and cortical neurons is known to bind TrkA and p75NTR to form a trimeric complex with NGF leading to neuronal survival pathways [6]. Upon binding to its receptors at the BFCN axon terminals, NGF signals retrogradely through vesicle and non-vesicle mediated transport mechanisms, thus supporting cholinergic physiology and activating survival signals [7,8,9,10,11,12,13,14]. In particular, in the vesicle-mediated transport the signaling complex of NGF is retrogradely transported through Rab5 positive “signaling endosomes” where NGF is associated with activated TrkA and downstream signaling proteins, like phospholipase Cγ (PLCγ), PI3K/Akt, MEK/ERK, suggesting that the activation of its pathway may occur already in these organelles [15,16,17]. In the case of reduced levels of mature NGF and/or TrkA, p75NTR preferentially binds the precursor form of NGF (Pro-NGF), and upon interaction with sortilin, transduces an apoptotic signaling responsible for AD neurodegeneration [18,19]. Interestingly, a higher Pro-NGF level has been found in AD brain tissues, and the imbalance between the two pathways is suggested to be a strong driver of the disease [20,21,22]. Along this lines, suppression of NGF/TrkA signaling in aged rats is associated to a marked impairment of cholinergic function and attentional performances [4,23,24,25,26]. In humans, a decrease in *TrkA* gene expression occurs during the progression from no cognitive impairment (NCI) to mild cognitive impairment (MCI), and from MCI to frank AD [27,28]. Impaired NGF signaling is linked to extensive loss of central cholinergic functions [29,30], a link that is supported by both cellular and animal models of AD [31,32,33]. Altogether, these findings suggest that NGF represents an important variable with regard to normal versus AD aging, an idea that led to recent clinical trials in which NGF replacement therapy was tested as a treatment for AD [34,35]. In particular, NGF gene therapy for AD patients was shown to induce a long-lasting trophic response and axonal sprouting in degenerating neurons in the absence of side-effects [36]. Moreover, NGF has a marked influence on synaptic vesicle exocytosis from BFCN presynaptic terminals [37]. This observation strongly supports the notion that alterations in NGF/TrkA signaling in these neurons could promote the synaptic failure and neurotransmission deficits associated with aging and AD-related cognitive impairment [4,35,38]. More explicitly, the so-called “neurotrophic model” hypothesizes that reduced availability of NGF and/or increased level of Pro-NGF, drives sporadic AD by linking the characteristic histopathological signs—such as synaptic pathology, cerebral Aβ deposits, neurofibrillary tangles, and memory loss—into a common neurodegenerative cascade [8]. In support of this hypothesis, initial results on the effects of imbalanced NGF/TrkA signaling on pathological amyloid precursor protein (APP) metabolism [39,40,41] were subsequently extended at the molecular level. Thus, transgenic mice that lack the APP-TrkA interaction [42], due to knock-in of the APP^YG/YG^ allele mutating Tyr682 to Gly, show marked degeneration of cholinergic neurons with related cognitive deficits. This suggests that reduced APP-TrkA binding could provide a good correlate of AD pathology and not just a general marker of neurodegeneration [43].

Furthermore, the APP/TrkA interaction is specifically lost in AD, but not in other neurodegenerative diseases such as Huntington’s disease (HD). Notably, APP/TrkA binding is severely affected only in AD target tissues, like the hippocampus, while other brain areas like the cerebellum are more resilient to neurodegeneration [44]. Moreover, NGF exposure increases the association between endogenous APP and TrkA in cultured septal neurons; in contrast, the association is disfavored by several agents known to induce cell death, such as Aβ, staurosporine, and rapamycin. These agents cause the dissociation of APP/TrkA complexes and increase the production of a C-terminal fragment of APP (CTFβ) [45]. Taken together, these results and observations suggest a model in which a deficit in NGF support leads to the first steps in degeneration of the BFCN. This may also trigger the Aβ pathology that in turn, spreads trans-synaptically to the neocortex and hippocampus [8,46,47,48]. Alternatively, an accumulation of independently-generated Aβ peptide may compromise the viability of TrkA-expressing neurons. This could lead to inhibition of NGF signaling and, then, in a negative feedback loop, to the onset of AD neuropathology [49,50].

Based on recent findings, which pinpoint the pathogenic role of the pro NGF-p75NTR/sortilin pathway in AD neurodegeneration, it may be predicted that a prevailing Pro-NGF signaling system in absence of TrkA [51,52,53] will increase JNK activity, APP^pT668^ levels, impair APP–TrkA interaction, and generate Aβ. Of interest, and adding a further level of complexity, Pro-NGF is able to downregulate TrkA via PTEN activation in brain neurons [54]. These issues are of foremost relevance for the complete understanding of APP metabolism in early AD and certainly deserve further investigations.

In this review, we focus on the control of cholinergic neurons metabolism by the NGF/TrkA system, with the long-term goal of identifying new approaches to improve the resilience of the cholinergic system to ageing and age-related neurodegeneration. In more detail, we highlight the dynamic interplay between the NGF/TrkA complex and APP processing in cholinergic neurons. The novel insights that emerge support the hypothesis that deficits in NGF/TrkA signaling, likely as a consequence of Pro-NGF/p75NTR/ sortilin signaling, could operate as a trigger of SAD [8,41,55,56]. This model is supported by the details of several molecular interactions. First, the metabolism and function of the APP interactome are shaped by the phosphorylation state of two APP motifs, ^667^VTPEE^671^ and ^682^YENPTY^687^ [57,58], and this phosphorylation is tightly controlled by NGF/TrkA signaling in hippocampal and cholinergic neurons, respectively [43,44]. Secondly, TrkA receptor binding to APP depends on a stretch of ~20 aminoacids in the juxtamembrane/extracellular domain of APP, and this precise region contains the α- and β-secretase cleavage sites. Importantly, defects in the NGF/TrkA pathway(s) are the cause of early synaptotoxicity, changes in neurotransmission, and alterations in tau metabolism. Taken together, with other findings from other laboratories add further support to a unifying hypothesis linking NGF pathophysiology with cholinergic degeneration, tau, and Aβ misfolding in the onset of SAD [55,59].

## 2. The Close Association of APP and TrkA

Accumulating evidence highlights a tight functional connection between the trafficking and signaling of NGF receptors and the processing and signaling of APP, implying a physical interaction between APP and NGF receptors. Studies by protein-fragment complementation assay (two hybrid assay) indicate that a very close spatial proximity between APP and p75NTR is required to allow a likely direct interaction between the two proteins [60]. By contrast, APP and TrkA interaction has been evaluated by co-immunoprecipitation, cross-linking, and colocalization approaches [42,44,61]. The most convincing data about the operational connection and a close physical interaction between TrkA receptor and APP specific residues has been provided by studies carried out with bimolecular fluorescence complementation (BiFC) and proximity ligation assay (PLA) [45]. These techniques allow the identification of interacting proteins close to each other over 7 and 15 nm distance, respectively. BiFC assay and co-immunoprecipitation of selected TrkA and APP deletion mutants in transfected HEK293 cells, allowed the mapping of the interacting domains involved in APP/TrkA complex. In particular, APP juxta-membrane region comprised between β- and α-secretase cleavage sites (amino acid position 597–613 numbering of the APP695 isoform, corresponding to the first 16 amino acid of Aβ) is sufficient to mediate interaction with TrkA. Interestingly, the same residues in APP (597–613) are also important for binding to p75NTR [60]. On the other side, the juxtra-transmembrane of TrkA is critical for interaction with APP while the intracellular domain of TrkA contributes to the binding to APP, likely by modulating the phosphorylation state of APP.

Obviously, such close vicinity between TrkA and APP—in the range of 10–15 nm—does not imply a direct interaction. Several proteins can be brought in proximity to each other by a partner that functions as a scaffold for the assembly of a trimeric protein complex. Simultaneous binding by two proteins in the vicinity of each other on the same scaffold might result in strong BiFC or PLA signal. However, co-expression of APP and TrkA BiFC plasmids with p75NTR, ShcC, and Mint-2-known shared binding partners paradoxically results in reduced BiFC signals. This finding suggests that neither p75NTR, nor ShcC, nor Mint-2 act as a bridging molecules between TrkA and APP [45]. ShcC and Mint-2 are cytosolic adaptor proteins that bind to the C-terminal cytosolic phospotyrosine motif of APP [62,63]. ShcC has been reported to influence APP traffic and processing by modulating the phosphorylation state of the ^667^VTPEE APP domain [44]. Mint-2 may regulate APP vesicular trafficking by serving as coat proteins [64]. These finding suggest that if other shared partners may be involved in TrkA/APP complex they most likely reside in other compartments or interact with another domain of APP. Example of the latter include sortilin, a type I neurospecific protein that acts as a receptor of neurotrophic factors and neuropeptides, and as co-receptor of G-protein coupled receptors, tyrosine receptor kinases, and ion-channels. Sortilin can also mediate trafficking from the secretory pathway to endosomes, as well as retrograde transport to the *trans*-Golgi network (TGN) after internalization from the cell surface [65]. Indeed, in cultured dorsal root ganglia, sortilin and TrkA interact via their extracellular domains to enhance anterograde transport and neurotrophin signaling [66]. Sortilin also interacts with the APP at both N- and C-terminal regions to influence both production and cellular uptake of APP [67]. Despite this physiological role, sortilin plays a key role also during neurodegeneration. As already mentioned, sortilin acts as a cell-surface co-receptor with p75NTR to mediate Pro-NGF-induced pro-apoptotic signaling [18,68] that occurs in conjunction with Aβ accumulations in AD. This action is not linked to increased expression of sortilin as its expression remains stable during the progression of AD [69,70] in the face of its upregulation in vitro by Aβ and p75NTR [71]. Its involvement in neurodegeneration is likely to reflect the new scenario occurring during AD, in which stable p75NTR, increased Pro-NGF, and reduced TrkA levels promote the formation of p75NTR/Pro-NGF death complex comprising sortilin.

Although further studies are required to evaluate a possible bridging role of sortilin or other unknown protein(s) for connecting APP and TrkA, the finding that APP and TrkA form an assembly mainly circumscribed to the plasma membrane, endoplasmic reticulum (ER), Golgi, and endocytic vesicle appears to be intriguing [45]. This distribution, evidenced by co-localization with compartment-specific markers both by BiFC analysis (in HEK-293 cells expressing exogenous APP and TrkA) and by PLA assay [in rat primary septal neurons cultivated for 10 days in vitro (DIV) in neurobasal plus B27 nutrient, expressing physiological level of APP and TrkA] was confirmed by treatment with drugs that perturb cellular trafficking [45], consistently with the known physiological transport of APP and TrkA in these compartments [72,73,74]. These findings suggest that the reciprocal influence both on the physiology and pathophysiology of the two proteins might occur in these compartments where both APP and TrkA form homodimers in the ER/Golgi before reaching the cell surface. It is interesting to note that in primary septal neurons: (i) NGF increases the number of TrkA/APP complexes in every compartment, including the ER and Golgi apparatus within 1 h of NGF treatment [45]; (ii) this increase is not the consequence of an augmented level of TrkA and APP [45] suggesting that, as mentioned below, this neurotrophin controls the level of APP/TrkA association by regulating the phosphorylation state of APP [44]. Conversely, TrkA/APP complexes decrease in number without any apparent degradation of TrkA or APP and before the loss of cell viability following NGF removal or cell death induced by Aβ peptide, staurosporine or rapamycin treatments [45]. This aspect is of particular relevance given that single cell gene array studies of BFCN did not reveal any changes in APP and APP related genes during the progression of AD [75]. Such death promoting agents, albeit acting via different mechanisms, make TrkA/APP complexes sensitive to cell death stimuli associated with amyloidogenic APP processing, which also occurs in NGF-deprived primary hippocampal neurons [39]. Since amyloidogenic APP processing is favored by APP/APP oligomerization, we hypothesize that TrkA binding to APP reduces the number of APP homodimers in physiological conditions and under NGF treatment and that, as suggested by preliminary experiments, during cell death and neurodegeneration, the dissociation of APP/TrkA complex allows APP to form homodimers which are more prone to processing by β- and γ-secretase.

## 3. The NGF/TrkA-ShcC Pathway Modulates APP Trafficking/Processing by Controlling Its Phosphorylation at the ^667^VTPEE Domain

Increasing evidence points to a critical role of post-translational modifications of the APP molecule in determining its amyloidogenic processing and shaping its signaling protein interaction networks including TrkA, shc, and Grb [57,58]. The APP modification most strongly implicated in amyloid generation and AD neuronal pathology is the phosphorylation of the threonine residue 668 (APP^pT668^) in the ^667^VTPEE domain of its intracellular tail [57]. APP^pT668^ has been shown to be neuron specific, to accumulate in dystrophic neurites in AD, and to strongly induce amyloid production and synaptic deficits, being proapoptotic in neurons [58,72,73,74,76]. According to the neurodegenerative role proposed for APP^pT668^, we found that APP^pT668^ halts APP binding to TrkA, and concomitantly increases the generation of CTF and Aβ in cholinergic neurons in vitro and in vivo [44]. Furthermore, APP phosphorylation at T668 promotes APP cleavage by caspases between residues Asp664 and Ala665, and generation of the cytotoxic AICD-C31 fragment involved in AD pathogenesis [77]. Similarly, the inhibition/reduction of APP^pT668^ levels in the brain may be a potential target for AD therapy [45]. Interestingly enough, our recent findings demonstrated that NGF is able to rapidly down regulate APP^pT668^ level in cultured BFCN as well as in septo-hippocampal slices [44].

The mechanism underlying reduced APP–TrkA interaction upon APP phosphorylation is not well understood. However, it is conceivable that APP phosphorylation prevents APP binding to TrkA by shuttling APP to TrkA-poor subcellular compartments. In fact, APP phosphorylation at T668 is associated with different intracellular localization, as compared to non-phosphorylated APP molecules [73]. To address this, we performed TrkA and APP^pT668^ double immunofluorescence stainings and observed that, indeed, TrkA and APP^pT668^ localize to the same subcellular compartments [44]. Instead, it is also possible that a conformational change in the APP molecule upon phosphorylation at T668 accounts for TrkA detachment from APP. This mechanism has been already described to occur with another APP interactor, Fe65 [78], whose detachment from APP dramatically increases neuronal Aβ production [79]. Thus, according to our findings, a conformational change of APP is more likely to explain the reduced APP–TrkA interaction observed in cholinergic neurons upon T668 phosphorylation of APP.

Assuming that the NGF signaling and APP metabolism are tightly connected, what are the specific molecular steps of such interplay? With the use of co-immunoprecipitation techniques and confocal imaging we found that (in rat primary septal neurons cultivated for 10 DIV in neurobasal plus B27 nutrient and in acute septo-hippocampal brain slices,) NGF stimulates TrkA binding to APP at the expense of APP binding to and cleavage by the β-secretase1 (BACE1), resulting in reduced generation of CTF and Aβ in cholinergic neurons [44]. Also upon NGF administration APP is trafficked to the Golgi system, where its binding to BACE1 is not favored in cholinergic neurons. In fact, total BACE_1_ levels are lower in the Golgi of cholinergic neurons, as compared to subcellular compartments like endosomes, where BACE_1_ is predominantly present in healthy cholinergic neurons [80]. Since NGF treatment stimulates APP binding to TrkA [44,45] and APP–TrkA complex has been observed in Golgi by PLA in cholinergic neurons [45], it can be hypothesized that TrkA mediates the trafficking of APP to the Golgi, similar to that described for another APP-shuttling molecule, named SorLA. SorLA is a sortilin-related endocytic receptor that belongs to the vacuolar protein sorting 10 (VPS10) domain receptor family. SorLA has been strongly implicated in APP trafficking in neurons and its reduction has been linked to sporadic AD in genome-wide association study (GWAS) studies [81]. Interestingly, the same APP point mutation (YG) is associated with loss of APP binding both TrkA [43] and SorLA [82], resulting in altered APP trafficking and increased amyloidogenesis in the forebrain neurons of APP^YG^ transgenic mice. In agreement with our model, this point mutation lies in the ^681^GYENPTY endocytic sorting signal [83] which drives APP re-routing to the trans-Golgi network (TGN), thus blocking the physiological trafficking/processing of APP. Accordingly, we observed that a reduced APP binding to and cleavage by BACE takes place in the Golgi system, where APP accumulates following NGF stimulation. Overall, these findings strongly pinpoint the key role of specific residues of the APP c-tail in regulating its binding to different intracellular players and consequent trafficking to BACE-enriched or poor neuronal compartments, thus finally affecting β fragments (CTFβ and Aβ) generation.

Also, the early adaptors and the downstream kinases implicated in the NGF-driven control of APP phosphorylation at T668 have been investigated. The data obtained indicate that the activation of the NGF pathway reduces APP^pT668^ levels by inhibiting the p54 kDa isoform of the ser/thr c-Jun N-terminal kinase (JNK), a well-known APP kinase in AD [44], through the activation of the TrkA-Sh2 containing sequence C (ShcC) signaling pathway. ShcC is a neuron-specific tyrosine kinase early adaptor, it mediates sustained survival signals in mature neurons upon neurotrophic stimulation, and is neuroprotective in vivo [84,85]. Docking of ShcC to TrkA upon NGF binding is responsible for the subsequent activation of the phosphoinositol-3 kinase (PI3K), a master inhibitor of the JNK pathway [86,87]. In turn, *shcC* deletion hinders APP-TrkA binding and elevates CTFβ and Aβ levels in BFCN [44], inducing typical signs of neuro-inflammation, cholinergic degeneration, and recognition memory deficits (preliminary data).

Altogether, the findings here summarized demonstrate that the NGF-TrkA/ShcC signaling pathway accounts for control of basal APP metabolism in mature cholinergic neurons of the mammalian brain. NGF preserves cholinergic neurons from pathological generation of Aβ by down-regulating APP phosphorylation at T668, allowing APP-TrkA binding and consequent APP trafficking to BACE-depleted subcellular compartments. NGF control of APP processing and downstream events described above are potentially disrupted in the human AD brain, as confirmed by recent findings on rodents and human pathology [19,88] confirming the pathologic significance of the APP-TrkA complex dissociation in the mammalian brain, and in particular in AD.

## 4. Impairment of NGF/TrkA Signaling Triggers an Early Activation of “a Dying-Back” Process of Degeneration of Cholinergic Neurons

Synaptic dysfunction is an early event in AD pathogenesis and is directly related to progressive cognitive impairment [89,90]. A large body of evidence indicates that neurons affected in AD follow a “dying-back pattern” of degeneration, where abnormalities in synaptic function and axonal connectivity long precede somatic cell death [91]. In fact, the early cognitive deficits occur during the AD progression in parallel with cortical synaptic loss [92,93,94,95] and are subsequently followed by death and/or atrophy of BFCN. The latter more directly accounts for the full-blown clinical symptoms of the disorder [38,96,97,98,99,100]. In view of the notion that, the synaptic density correlates more closely with memory/learning impairment than any other pathological lesion observable in the AD neuropathology [101] and that dysfunction of NGF/TkA signaling underlies the selective degeneration of cortical cholinergic projecting neurons in AD pathogenesis [29], septal primary cultures have been recently employed by our research group with the intent of analyzing the NGF activity and consequences, at the synaptic level, of its in vitro withdrawal. In order to sensitize primary neurons to the following removal of trophic factor, we have recently developed a novel culturing procedure whereby pretreatment (10 DIV) with NGF in the presence of low 0.2% B27 nutrients, selectively enriches (+36%) NGF-responsive forebrain cholinergic neurons at the expense of all other non-cholinergic resident populations, such as GABAergic (~38%) and glutamatergic (~56%) [102]. This simple, less expensive but valuable method allows a consistent and fully mature cholinergic population to be obtained, as demonstrated by biochemical, morphological, and electrophysiological approaches. In fact, this culturing procedure can actually represent an important achievement in the research of AD neuropathology since the yield in cholinergic neurons following the classical culture protocols is lower [103,104,105,106]. By taking advantage of this newly-established in vitro neuronal paradigm, we revealed that the NGF withdrawal induces a progressive deficit in the presynaptic excitatory neurotransmission which occurs in concomitance with a pronounced and time-dependent reduction in several distinct pre-synaptic markers, such as synapsin I, SNAP-25, and α-synuclein, and in the absence of any sign of neuronal death. This rapid presynaptic dysfunction: (i) is reversible in a time-dependent manner, being suppressed by de novo external administration of NGF within six hours from its initial withdrawal; (ii) is specific, since it is not accompanied by contextual changes in expression levels of non-synaptic proteins from other subcellular compartments including specific markers of endoplasmic reticulum and mitochondria such as calnexin, VDAC, and Tom20; (iii) is not secondary to axonal degeneration, because it precedes the post-translational modifications of tubulin subunits critically controlling the cytoskeleton dynamics and is insensible to pharmacological treatment with known microtubule-stabilizing drug such paclitaxel; (iv) involves TrkA-dependent mechanisms because the effects of NGF re-application are blocked by acute exposure to a specific and cell-permeable inhibitor of TrkA receptor. In addition, in line with previous findings reporting a modulatory effect of NGF signaling on APP expression [39,107], a significant upregulation in expression of three APP isoforms of ~110 kDa, ~120 kDa, and ~130 kDa along with marked increase in the immunoreactivity level of the carboxyl-terminal CTFβ fragment of 14 kDa, are detected in primary septal neurons upon 24–48 h of neurotrophin starvation, indicating that the APP metabolism is also greatly influenced following NGF withdrawal in this novel AD-like neuronal paradigm. These results clearly demonstrate that the combined modulatory actions of NGF on the expression of important pre-synaptic proteins and neurosecretory function(s) of in vitro cholinergic septal primary neurons are directly and causally linked via the stimulation of NGF/TrkA signaling. These findings point out the pathological relevance of the lack of NGF availability in the earliest synaptic deficits occurring at the onset of AD progression [102]. Taken together, these findings: (i) provide a valuable in vitro tool to better investigate the survival/disease changes of basal forebrain septo-hippocampal projecting neurons occurring during the prodromal stages of AD pathology caused by dysfunction in NGF/TrkA signaling; (ii) demonstrate that NGF withdrawal induces neurodegenerative changes initiated by early, selective, and reversible presynaptic dysfunction in cholinergic neurons, just resembling the synapses loss and retrograde “dying-back” axonal degeneration appearing at prodromal stages of AD pathology in correlation with incipient memory dysfunction [46,108,109,110]; (iii) have potential, important clinical implications in the field of therapeutical in vivo NGF delivery in humans, because it not only constitutes a molecular rationale for the existence of its limited therapeutic time window, but also offers a prime useful presynaptic-based target with the intent of extending its neuroprotective action in AD intervention.

## 5. Impaired NGF Signaling in AD Synaptic Failure: Crucial Role of N-Terminal Cleavage of Tau Protein

The amyloid hypothesis [111,112] suggests that Aβ is upstream of tau in AD pathogenesis by triggering its conversion from normal into toxic conformation. The validity of the amyloid hypothesis has been questioned after the failure of Aβ targeting therapy in clinical trials [113]. Of note, the insufficient target engagement, the late drug administration in the disease process, and the new emerging pathological Aβ species are important causative factors contributing to disappointing results from Alzheimer’s trials. A more integrative model of AD-like neurodegeneration has been also proposed in view of the fact that tau pathology without amyloidosis is a major constituent of suspected non-Alzheimer disease pathophysiology (SNAP) and subcortical tau might be the initial pathological event, with amyloid pathology developing independently of it, and possibly accentuating, tauopathy [114,115]. Although it is premature to write off amyloid hypothesis, many studies indicate a synergism between plaques and tangles [116,117,118,119,120] with abnormal tau enhancing Aβ peptide toxicity via a negative feedback loop [112] or vice versa [114,115].

Among abnormal posttranslational modifications of tau, cleavage on N-terminal extremity occurs early in onset/progression of AD and not-AD tauopathies [121,122,123,124]. Importantly, the *N*-truncated form(s) lacking the microtubule binding domains plays a not-dispensable role in AD etiopathology [125,126,127,128,129], mainly at synaptic terminals [130,131]. Indeed, passive immunotherapy with antibody targeting the N-terminal projection domain of human tau has been proved to be beneficial in improving cognitive deficits in AD transgenic mice [132,133,134] suggesting that the targeting of pathological tau is a promising disease-modifying cure for tauopathies in the near future. Consistent with the crucial pathogenetic role of tau in AD, NGF has been demonstrated to regulate the steady-state levels [135] and the post-translational modification of tau, including phosphorylation, cleavage, and ubiquitination [136,137,138,139,140]. Axonal degeneration and cytoskeleton disruption, which are both early features of AD and other neurodegenerative dementias [141], are associated with an imbalanced distribution and dysregulation in NGF/TrkA signaling [142], due to the loss of microtubule-binding capacity and/or accumulation of tau into the somato-dendritic compartment. To this regard, we have reported that NGF withdrawal leads to Aβ-dependent hyperphosphorylation and truncation of this protein in hippocampal neurons with production of diagnostic 20–22 kDa NH_2_ fragment [139]. This NH_2_-derived truncated form of tau-mapping between 26 and 250 amino acids of the longest human tau isoform (htau40) has been also detected in animal AD models characterized by an impaired NGF signaling [143] and in human AD synaptosomes in tight correlation with the synaptic changes, Aβ deposition and pathological tau alterations (phoshorylation/aggregation/misfolding) [144,145]. Furthermore, this peptide is also preferentially released from AD presynaptic terminals into parenchyma [146] and is detectable in cerebrospinal fluid (CSF) from living patients affected by AD and other neurodegenerative diseases associated with dementias [147] suggesting that its dynamic evaluation in CSF in the ordinary clinical practice can be exploited for diagnostic/prognostic approaches in AD treatment. In view of the notion that deregulation in NGF/TrkA signaling favors the amyloidogenic processing in affected neurons [39,42,44] and that tau reduction prevents the Aβ induced defects on synapses integrity and functions [123,125], these findings appear particularly relevant for tau physiopathology in the field of AD and other tauopathies, helping to develop a best-targeted and more effective tau-based immunotherapy based on the depletion of intracellular/extracellular toxic tau species [148,149].

## 6. Conclusions

**Figure 1:**
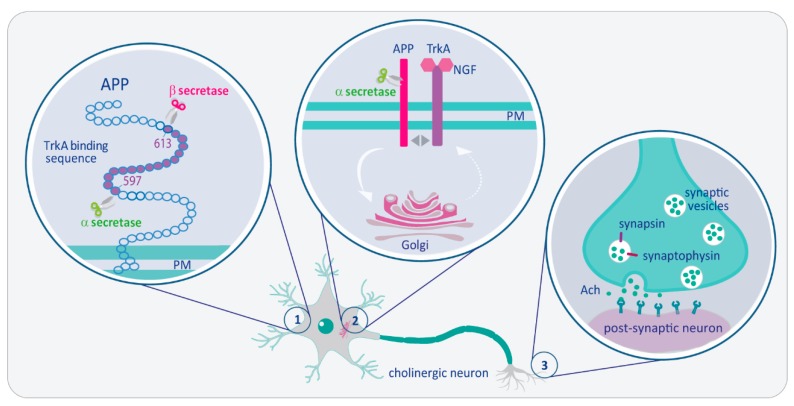
NGF/TrkA pathway modulates Amyloid Precursor Protein (APP) metabolism and synaptic functions in adult forebrain cholinergic neurons. (1) Studies reported in this review and in former studies indicate that the stretch of 16 amino acids (597–613) of the juxtamembrane region of APP-695 molecule (human APP numbering) contains also the binding sites for the NGF receptors TrkA and p75, as well as the cleavage sites for α- and β-secretase; (2) Such tight proximity might allow NGF-TrkA complex to stimulate α-secretase activity in the plasmamembrane (PM) and APP trafficking from the PM to the Golgi (arrow), and most probably back to PM (dashed arrow), also enriched of α-secretase. By recycling APP through neuronal compartments where β-secretase is poorly represented, NGF ensures the preferential anti-amyloidogenic cleavage of APP by the α-secretase; (3) At the synaptic compartment, NGF/TrkA pathway sustains the expression level of synaptic vesicle proteins, like synapsin and synaptophysin, and the consequent Ach release, thus subserving physiological synaptic functions in cholinergic neurons. (BFCN, basal forebrain cholinergic neurons; APP, Amyloid precursor protein; PM, plasmamembrane; Ach, acetylcholine). The illustration was realized by V.T., with the help of Isabella Triaca for graphic elaboration.

As briefly depicted and summarized in Figure 1, studies carried out both in our and other laboratories underline the potentially tight structural and functional interaction among APP, α- and β-secretase, on the one side, and the high (TrkA) and low (p75NTR) affinity NGF receptors on the other side. This interplay deals with APP as a unique source of both “good” peptides (sAPPα), produced by α-secretase, and a “bad” peptide, generating Aβ. Although the functional significance of the “good” peptide is still the object of several studies, the noxious action of Aβ as a trigger of the onset of Alzheimer’s is widely acknowledged. The fact that biological functions of these important molecular players require this highly conserved stretch of 16 amino acids, out of the large (ca. 1000 amino acids) APP molecule, pinpoint to a potentially crucial physiological significance.

The largely documented role of NGF-TrkA complex in this scenario, summarized in this review and in other studies, points to NGF as an actually potent therapeutic agent in AD and has implications for the pathological effect of its reduced supply to basal forebrain cholinergic neurons.

## Abbreviations

NGF	Nerve growth factor
TrkA	High-affinity tropomyosin receptor kinase A
p75NTR	Neurotrophin receptor p75
BFCN	Basal forebrain cholinergic neurons
AD	Alzheimer’s disease
APP	Amyloid precursor protein
MCI	Mild cognitive impairment
BiFC	Bimolecular fluorescence complementation
PLA	Proximity ligation assay
BACE1	β-Secretase 1
Aβ peptide	Amyloid β peptide
JNK	Ser/Thr c-Jun N-terminal kinase
ShcC	Sh2 containing sequence C
PI3K	Phosphoinositol-3 kinase
TGN	*Trans*-Golgi network
ER	Endoplasmic reticulum
